# Psychosocial factors associated with pain and health‐related quality of life in Endometriosis: A systematic review

**DOI:** 10.1002/ejp.2006

**Published:** 2022-07-22

**Authors:** Michail Kalfas, Claudia Chisari, Sula Windgassen

**Affiliations:** ^1^ Health Psychology Section Institute of Psychiatry, Psychology, and Neuroscience, King's College London London UK

## Abstract

**Background and Objective:**

Endometriosis is associated with pain and impaired health‐related quality of life (HRQoL). Psychosocial factors have been associated with pain and HRQoL in other conditions, suggesting their potential relevance in Endometriosis. However, the role of psychosocial factors in this population has not been systematically explored yet. This systematic review aims to explore the association of psychosocial factors with pain intensity/severity and HRQoL in women with Endometriosis.

**Databases and Data Treatment:**

Observational and experimental studies that explored the association of psychosocial factors with pain and HRQoL in women with Endometriosis were eligible. The following databases were searched: Medline, Embase, Cochrane library, Web of Science, PsychInfo and Cumulative index of nursing and allied health literature. The methodological quality was assessed, and findings were synthesized using narrative synthesis.

**Results:**

Twenty‐seven studies were eligible for inclusion, which included 5419 women with Endometriosis. Catastrophising and anxiety were the factors most consistently associated with greater pain, whilst depression, anxiety and stress were related to worse HRQoL. Findings regarding depression and pain were mixed, and research on social factors was limited.

**Conclusions:**

This systematic review highlights the role of psychosocial factors in Endometriosis. Anxiety, depression and catastrophising are suggested as potential treatment targets. The review also indicates the lack of research on other potentially important psychosocial factors, such as avoidance, perceived injustice and social support.

**Significance:**

This systematic review explored the role of psychosocial factors in Endometriosis, suggesting that these are associated with pain and health‐related quality of life (HRQoL). Among the psychosocial factors included, anxiety, depression and catastrophising were the factors most often associated with pain and HRQoL in Endometriosis. These findings highlight the need to target psychological factors in the treatment of women with Endometriosis.

## 1 INTRODUCTION

Endometriosis is a gynaecological condition in which endometrial cells grow in the surrounding areas outside the uterus, such as the ovaries and fallopian tubes (Farquhar, 2007). The prevalence of Endometriosis is around 10% in reproductive‐age women (Rogers et al., 2009); however, the prevalence in infertile women can reach 40% (Meuleman et al., 2009). The primary symptoms of Endometriosis include chronic pelvic pain, dysmenorrhea (menstrual pain), dyspareunia (pain during sexual intercourse), dysuria and dyschezia (painful urination and defecation) and infertility (Ballard et al., 2008; Farquhar, 2007).

Recent meta‐analyses have demonstrated that Endometriosis has a negative impact on mental health and quality of life, similar to that of chronic pain (Van Barneveld et al., 2022; Wang et al., 2021). Pain in Endometriosis is associated with greater depression and anxiety (Gambadauro et al., 2019; Pope et al., 2015), as well as poorer health‐related quality of life (HRQoL) (Jia et al., 2012). Pain is a central symptom in Endometriosis as 64.2% of women with this condition report pelvic pain or dysmenorrhea (Ballard et al., 2008). Surgical removal of scar tissue has been used as a treatment option in Endometriosis; however, 20% of women with Endometriosis do not report pain improvement, and for some, the pain temporarily decreases but eventually returns (Abbott et al., 2004). Research in persistent pain demonstrates the influential and interactive role of psychosocial factors on pain, indicating that pain is better understood as a biopsychosocial experience (Gatchel et al., 2007). Systematic reviews in persistent pelvic pain and Vulvodynia have shown that psychosocial factors are associated with greater pain and worse quality of life (Chisari, Monajemi, et al., 2021; Riegel et al., 2014,), and they have also suggested that different psychosocial factors may be operating across different conditions. In Endometriosis, depression (Facchin et al., 2016), anxiety (Facchin et al., 2017; Lagana et al., 2017) and catastrophising (Martin et al., 2011) have been associated with greater pain.

Women with Endometriosis often experience stigma, invalidation and dismissal from health professionals (Cox et al., 2003; Lamvu et al., 2020; Sims et al., 2021). These experiences not only have an impact on the diagnosis of this condition, which takes 8–9 years on average (Ghai et al., 2020; Tewhaiti‐Smith et al., 2022) but also increase psychological distress. For instance, feelings of pain invalidation by medical professionals have been shown to increase shame and depressive symptoms (Boring et al., 2021). Understanding the role of psychosocial factors in relation to outcomes in Endometriosis could raise awareness among healthcare professionals about their importance in the conceptualisation and management of this condition; this, in turn, can contribute to a shift from a biomedical view of the condition to a broader conceptualization that is more comprehensive. To support this, previous research testing psychological interventions in Endometriosis has shown significant improvements in participants' quality of life (Kold et al., 2012; Van Niekerk et al., 2019). To date, no systematic reviews have been conducted to examine the association between psychosocial factors, pain and HRQoL in Endometriosis. A systematic review investigating psychosocial factors could provide greater insight into the role of these variables in the context of Endometriosis, thus furthering biopsychosocially informed research and healthcare.

This systematic review aims to explore the association of psychosocial factors with (1) pain intensity and severity and (2) HRQoL in women with Endometriosis.

## 2 LITERATURE SEARCH METHODS

### 2.1 Protocol

This systematic review was designed in accordance with the Preferred Reporting Items for Systematic Reviews and Meta‐Analyses (PRISMA) guidelines (Page et al., 2021a, 2021b). The systematic review protocol is available at: https://www.crd.york.ac.uk/prospero/display_record.php?RecordID=238130

### 2.2 Eligibility criteria

Eligible studies had a quantitative observational or experimental design and explored the association, through correlation or regression analyses, of psychosocial factors with pain intensity and severity, and HRQoL in women with Endometriosis. Only studies published in English with full‐text available were included, and no publication date restrictions were applied. Women aged 16 years or older who had a confirmed diagnosis of endometriosis were included. This age limit was applied because all the female puberty milestones are reached by the age of 16 (Brix et al., 2019) and Endometriosis is most commonly diagnosed in women of reproductive age (i.e., 15–49) (Haas et al., 2012). Psychosocial factors refer to any cognitive (e.g., catastrophising), emotional (e.g., anxiety), behavioural (e.g., coping) and social factors (e.g., relationship status), as well as personality traits (e.g., neuroticism).

### 2.3 Search strategy and selection

**FIGURE 1:**
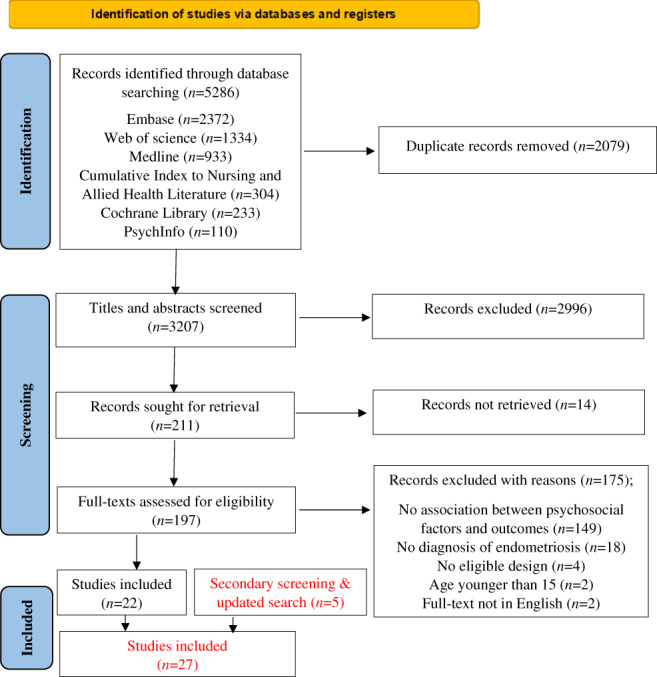
Study flow diagram according to PRISMA 2020 (Page et al., 2021a, 2021b).

A systematic search was conducted on Medline, Embase, Psychinfo, Cumulative index to nursing and allied health literature (CINAHL), Cochrane library and Web of Science in April 2021. The database search was performed using key terms for pain, HRQoL and psychosocial factors combined with the operators “OR” and “AND”. Various keywords were used to describe psychological (e.g., catastrophising) and social factors (e.g., social support), which were combined using the operator “OR”. Exploded terms and Medical Subjects Headings (MeSH) were used to maximize results. Publication date restrictions were not applied. The full search terms are presented in Table S1. Identified studies from the database search were assessed in three stages using the PRISMA 2020 flowchart for systematic reviews (Page et al., 2021a, 2021b). Articles were saved on EndNote X9 reference manager (Clarivate, 2013). These were then sorted alphabetically, and duplicates were removed. Titles, abstracts and full texts were screened initially by the first author (MK) using predefined eligibility criteria, and ineligible studies were excluded for reasons. Double screening was conducted by the other two co‐authors (SW and CC). The screening procedure is presented in the PRISMA 2020 flowchart (Figure 1) (Page et al., 2021b).

### 2.4 Data extraction and synthesis

Data from the included studies were extracted by MK. Extracted information included: study details (reference, country); study design; sample size; sociodemographic characteristics (age); outcome variable(s); outcome measure(s) (i.e., pain and HRQoL); psychosocial factor(s); psychological measure(s); statistical analysis; key findings. Findings of the systematic review were presented using narrative synthesis, according to the synthesis without meta‐analysis (SWiM) in systematic reviews (Campbell et al., 2020). Due to the heterogeneity of the psychosocial factors included and the fact that many studies did not report the effect sizes, a meta‐analysis was not performed.

### 2.5 Risk of bias assessment

The methodological quality and risk of bias of eligible full‐text articles were assessed using an adapted Critical Appraisal Skills Programme [CASP] checklist (CASP, 2019). Tailoring the quality assessment tool is recommended in the Cochrane Handbook (Higgins et al., 2011). The adapted checklist was developed by the authors and was based on the case–control and cohort‐study CASP checklists. It included eight questions with a three‐point response scale (i.e., “yes”, “no” and “cannot tell”). To assess the quality of studies, the “yes” answers (i.e., yes = 1) were added up. Scores from 0 to 2 were classified as low quality, scores from 3 to 5 as medium quality and scores from 6 to 8 as high quality. As this was an adapted scale, this tool did not undergo its own validation. The CASP checklist that was used is presented in Table S2.

### 2.6 Effect measures

The primary outcomes were pain intensity/severity, and HRQoL in Endometriosis. These were explored using associations (i.e., bivariate correlations and regression coefficients) between psychosocial factors and the outcome measures. Correlation coefficients were interpreted as small (*r* = 0.10), medium (*r* = 0.30) and large (*r* = 0.50). Similarly, the effect sizes of regression coefficients were classified as small (*f* ^2^ = 0.02), medium (*f* ^2^ = 0.15) and large (*f* ^2^ = 0.35). This classification was informed by Cohen's effect size guide (1992). The effect sizes for the regression were calculated using the *r* ^2^ coefficient, when available (Table 1).

**TABLE 1:** Inclusion and exclusion PICOS criteria (Page et al., 2021b)

	Inclusion Criteria	Exclusion Criteria
Population	Women	Men
	Age 16 years old or older	Age 15 years old or younger
	Clinical diagnosis of endometriosis (using laparoscopy, histological examination, ultrasound or magnetic resonance imaging)	No clinical diagnosis of endometriosis
Intervention	Psychological factors (e.g., emotional and cognitive factors); Social factors (e.g., dyadic factors); Personality factors (e.g., neuroticism)	Demographic characteristics (e.g., age, ethnicity)
Control/Comparison	‐	‐
Outcomes	Self‐report pain intensity/severity measured by a validated measure, a visual analogue or a numerical pain rating scale	Pain intensity/severity measured by a non‐validated psychometric questionnaire
	Disease‐related interference assessed by health‐related quality of life	General quality of life
Study Design	Quantitative, observational retrospective, prospective, case–control, cohort, longitudinal and experimental studies	Non‐empirical, qualitative or review papers, reports, books
	Studies published in English	Studies not published in English
	Accessible studies	Not accessible studies

## 3 RESULTS

The database search resulted in 5286 records. Following the removal of duplicates and the screening of title and abstract, 211 full‐text articles were assessed for inclusion. 27 studies were included in this systematic review. The number of studies included at each stage is presented in the PRISMA flowchart (Figure 1).

### 3.1 Overview of studies

All included studies were observational, and 26 of them were cross‐sectional. One study (Martin et al., 2011) was prospective and had a 12‐month follow‐up. A mixed‐method study was included (Zarbo et al., 2019), but only the quantitative data were extracted. The 27 included studies composed of 5419 participants in total. Two studies included the same sample (Melis et al., 2014, 2015). Overall, the age of participants ranged between 17 and 71 years old, and the mean age ranged between 30.5 and 36.7 years old. Nine studies took place in Italy, three in Brazil, two in the United States and two in Poland whilst the remaining 11 studies took place in various countries. One study (De Graaff et al., 2013) was multicentre and participants were recruited in 10 different countries. The characteristics of the included studies are presented in Table 2.

**TABLE 2:** Study characteristics

Reference	Country	Sample Size	Mean age (SD)	Study design
Andysz & Merecz‐Kot, 2021	Poland	*n* = 247	32.5 (6.2)	Cross‐sectional
Bylinka & Oniszczenko, 2016	Poland	*n* = 103	30.5 (4.8)	Cross‐sectional
Carey et al., 2014	United States	*n* = 79	36.4 (7.2)	Cross‐sectional
Cavaggioni et al., 2014	Italy	*n* = 37	35 (7.6)	Cross‐sectional
De Graaff et al., 2013	10 different countries	*n* = 931	36.1 (6.3)	Cross‐sectional
Eriksen et al., 2008	Denmark	*n* = 63	33.1 (7.3)	Cross‐sectional
Facchin et al., 2015	Italy	*n* = 110	‐	Cross‐sectional
Facchin et al., 2016	Italy	*n* = 82	‐	Cross‐sectional
Facchin et al., 2017	Italy	*n* = 210	36.7 (7.0)	Cross‐sectional
Facchin et al., 2019	Italy	*n* = 127	35.4 (7.4)	Cross‐sectional
Laganà et al., 2015	Italy	*n* = 166	36 (6)	Cross‐sectional
Márki et al., 2017	Hungary	*n* = 193	33.8 (5.37)	Cross‐sectional
Martin et al., 2011	United States	*n* = 115	‐	Prospective
Martins et al., 2021	Portugal	*n* = 124	36.1 (6.51)	Cross‐sectional
McPeak et al., 2018	Canada	*n* = 236	35 (7.3)	Cross‐sectional
Melis et al., 2014	Italy	*n* = 41	31.4 (6.4)	Cross‐sectional
Melis et al., 2015	Italy	*n* = 41	31.4 (6.4)	Cross‐sectional
Mińko et al., 2021	Poland	*n* = 484	33.1 (6)	Cross‐sectional
Mundo‐López et al., 2020	Spain	*n* = 230	36.7 (5.2)	Cross‐sectional
O'Hara et al., 2021	Australia	*n* = 620	34.6 (9.5)	Cross‐sectional
Petrelluzzi et al., 2008	Brazil	*n* = 93	33.8 (1)	Cross‐sectional
Petrelluzzi et al., 2012	Brazil	*n* = 26	32.2 (1.3)	Cross‐sectional
Roomaney et al., 2020	South Africa	*n* = 202	34.9 (7.04)	Cross‐sectional
Sepulcri & Amaral, 2009	Brazil	*n* = 104	34.6 (6.3)	Cross‐sectional
Sullivan‐Myers et al., 2021	Australia	*n =* 584	31.2 (7.5)	Cross‐sectional
van Aken et al., 2017	Netherlands	*n* = 50	34.5 (7)	Cross‐sectional
Zarbo et al., 2019	Italy	*n* = 162	‐	Cross‐sectional

Psychological factors were grouped as general distress (anxiety, depression, stress, generalized worry and somatisation), cognitive factors (catastrophising, beliefs, self‐efficacy, rumination, self‐esteem, illness acceptance), behavioural factors (self‐care activities), emotion regulation factors (emotion regulation and alexithymia), social factors (relationship status, presence of partner and marital satisfaction) and personality traits (character/temperament traits).

The included studies assessed pain and HRQoL. Pain was assessed using numerical rating, visual analogue scales or the short‐form McGill pain questionnaire (SF‐MPQ) (Melzack, 1975). Given that studies used different terms to quantify pain (i.e., pain intensity or severity), albeit they used the SF‐MPQ (Carey et al., 2014; Martin et al., 2011; Zarbo et al., 2019), the terms pain intensity and pain severity were considered synonymous in this review. HRQoL was measured using the Endometriosis health profile‐30 (EHP‐30) (Jones et al., 2001) or the short‐form health survey (SF‐36) (Ware Jr & Sherbourne, 1992). To explore these outcomes in relation to psychosocial factors, 14 studies used bivariate correlations (Pearson's *r* and Spearman *rho*), ten studies used simple or multiple linear regression, whilst three studies used both Pearson's *r* correlation and multiple linear regression.

### 3.2 Risk of bias

Twenty‐two studies were rated as medium methodological quality, and five studies as high quality (Andysz & Merecz‐Kot, 2021; De Graaff et al., 2013; Martin et al., 2011; Martins et al., 2021; O'Hara et al., 2021). The risk of bias assessment for each study is presented in Table S3.

## 4 MAIN RESULTS

The findings of included studies for pain and HRQoL are presented below and are also shown in Tables 3 and 4. Eight studies did not report the *r* ^2^ coefficient of the regressions, and thus, it was not possible to calculate the effect size. In two studies, the standardized regression coefficient *β* was not given, and therefore only the unstandardised *B* regression coefficients are presented in the results.

**TABLE 3:** Psychosocial factors associated with pain in Endometriosis

Reference	Psychological factor	Outcome	Analysis	Key results	Effect size	Quality score
Cognitive factors						
Bylinka & Oniszczenko, 2016	Beliefs about pain control [beliefs about pain control questionnaire]	Pain intensity [numerical rating scale‐11]	Pearson's *r* correlation Multiple hierarchical linear regression	Internal beliefs about pain (*r* = −0.31, *β* = −0.24, *p* < 0.01); Beliefs about doctor control of pain (*r* = 0.33, *p* < 0.01); Beliefs that pain was controlled by chance events (*p* > 0.05).	Medium	Medium
Carey et al., 2014	Catastrophising [coping strategies questionnaire]	Pain intensity [short form McGill pain questionnaire]	Simple linear regression	Affective pain (*β* = 0.66, *p* = 0.01); total pain score (*p* = 0.055).	‐	Medium
Facchin et al., 2019	Self‐esteem [Rosenberg self‐esteem scale]	Pelvic pain presence [categorical question]	Multiple hierarchical linear regression	*β* = −0.22, *r* ^2^ = 0.06, *p* < 0.01	Small	Medium
Facchin et al., 2017	Rumination [ruminative response scale]	Pain severity [10‐point numerical rating scale]	Multiple hierarchical linear regression	*β* = 0.25, *p* < 0.001	‐	Medium
Martin et al., 2011	Catastrophising [coping strategies questionnaire]	Pain severity [short form McGill pain questionnaire]	Multiple linear regression	*β* = 0.18, *p* = 0.04, *r* ^2^ = 0.03	Small	High
Zarbo et al., 2019	Catastrophising [cognitive emotion regulation questionnaire—short version] Self‐blame [cognitive emotion regulation questionnaire—short version]	Pain severity [short form McGill pain questionnaire]	Pearson's *r* correlation	*r* = 0.26, *p* < 0.001 *r* = 0.154, *p* < 0.001	Small	Medium
General distress						
Eriksen et al., 2008	Anxiety [state–trait anxiety inventory] Depression [beck depression inventory]	Pain severity [visual analogue scale]	Pearson's *r* correlations	*P*s >0.05	‐	Medium
Facchin et al., 2019	Anxiety Depression [hospital anxiety and depression scale]	Pelvic pain presence [categorical question]	Multiple hierarchical linear regression	*β* = 0.22, *r* ^2^ = 0.06, *p* < 0.01 *p* > 0.05	Small	Medium
Facchin et al., 2017	Anxiety Depression [hospital anxiety and depression scale]	Pain severity [10‐point numerical rating scale]	Multiple hierarchical linear regression	*β* = 0.21, *p* < 0.001 *β* = 0.19, *p* < 0.001	‐	Medium
Facchin et al., 2015	Anxiety Depression [hospital anxiety and depression scale]	Pain intensity (dysmenorrhea dyspareunia, dyschezia, non‐menstrual pelvic pain) [10‐point numerical rating scale]	Multiple linear regression	Non‐menstrual pain (*β* = 0.285, *p* < 0.01) Non‐menstrual pain (*β* = 0.47, *p* < 0.001); Dysmenorrhea, dyspareunia, dyschezia (*ps* >0.05)	‐	Medium
Laganà et al., 2015	Anxiety [self‐ rating anxiety scale] Depression [self‐rating depression scale] Somatisation [symptom checklist 90‐R]	Pelvic pain presence [categorical question]	Pearson's *r* correlations	*r* = 0.26, *p* < 0.01 *r* = 0.25, *p* < 0.01 *r* = 0.31, *p* < 0.01	Small Small Medium	Medium
Mińko et al., 2021	Anxiety Depression [hospital anxiety depression scale]	Pain during intercourse [female sexual function index]	Spearman's *rho* correlations	*r* = 0.20, *p* < 0.001 *p* > 0.05	Small	Medium
Petrelluzzi et al., 2012	Perceived stress [perceived stress questionnaire]	Pain intensity [10‐point visual analogue scale]	Pearson's *r* correlation	*p* > 0.05	Large	Medium
Sepulcri & Amaral, 2009	Anxiety [state–trait anxiety inventory; Hamilton rating scale for anxiety]	Pain intensity [10‐point visual analogue scale]	Pearson's *r* correlation	State, *p* < 0.01; trait, *p* = 0.048; Hamilton anxiety (*p* = 0.001)	‐	Medium
Zarbo et al., 2019	Generalized worry [Penn‐state worry questionnaire]	Pain severity [short form McGill pain questionnaire]	Pearson's *r* correlations	*r* = 0.32, *p* < 0.001	Medium	Medium
Emotion regulation						
Cavaggioni et al., 2014	Alexithymia [Toronto alexithymia scale]	Pain intensity [10‐point visual analogue scale]; dyspareunia [10‐point visual analogue scale]	Spearman's *rho* correlations	*r* = 0.36, *p* = 0.04. Alexithymia total score (*p* = 0.56); difficulty identifying feelings (*r* = −0.35, *p* < 0.05).	Medium	Medium
Personality						
Bylinka & Oniszczenko, 2016	Temperament traits [formal characteristics of behaviour‐temperament inventory]	Pain intensity [numerical rating scale‐11]	Pearson's *r* correlation Hierarchical linear regression	Endurance (*r* = −0.51; *β* = −0.56, *p* < 0.01); other temperament traits (*p*s >0.05).	High	Medium
Facchin et al., 2016	Character and personality traits [temperament and character inventory‐revised]	Pain severity [numerical rating scale‐10]	Multiple hierarchical linear regression	Harm avoidance (*B* = 0.08; *SE* = 0.02; *p* < 0.01); self‐directedness (*B* = ‐0.05; *SE* = 0.02; *p* < 0.05); other character/temperament traits (*p*s <0.05).	‐	Medium

**TABLE 4:** Psychosocial factors associated with health‐related quality of life in Endometriosis

Reference	Psychological factor	Outcome	Analysis	Key results	Effect size	Quality score
Cognitive factors						
Andysz & Merecz‐Kot, 2021	Illness acceptance [Acceptance of Illness Scale]	HRQoL [EHP‐30]	Hierarchical linear regression	HRQoL: pain subscale (*β* = −0.27, *p* < 0.001)	‐	High
McPeak et al., 2018	Catastrophising [Pain catastrophising scale]	HRQoL [EHP‐30]	Spearman *rho* correlation	*rho* = 0.56, *p* < 0.001	Large	Medium
Melis et al., 2015	Body attitude [body attitude test]	HRQoL [36‐item short‐form survey]	Spearman *rho* correlations	Negative appreciation of body size: HRQoL total score (*rho* = −37, *p* < 0.001); physical HRQoL (*rho* = −0.38, *p* < 0.001); mental HRQoL (*rho* = −0.31, *p* < 0.001). Lack of familiarity with own body: HRQoL total (*rho* = −59, *p* < 0.001); physical HRQoL (*rho* = −0.58, *p* < 0.001); mental HRQoL (*rho* = −0.49, *p* < 0.001). Body dissatisfaction: HRQoL total (*rho* = −43, *p* < 0.001); physical HRQoL (*rho* = −0.39, *p* < 0.001); mental HRQoL (*rho* = −0.35, *p* < 0.001).	Medium Large Medium Medium	Medium
Mundo‐López et al., 2020	Catastrophising [Pain catastrophising scale]	HRQoL [36‐item short form survey]	Multivariate regression	*β* = 1.47, *p* < 0.001	‐	Medium
O'Hara et al., 2021	Self‐efficacy [self‐efficacy for managing chronic disease]	HRQoL [36‐item short form survey]	Hierarchical linear regression	Physical HRQoL (*B* = 1.30, *p* < 0.001); mental HRQoL (*B* = 1.55, *p* < 0.001)	‐	High
van Aken et al., 2017	Pain cognition [pain catastrophising scale; pain vigilance and awareness questionnaire; pain anxiety symptoms scale]	HRQoL [36‐item short form survey; Endometriosis health profile‐30]	Pearson's *r* correlation Multivariate linear regression	EHP‐30 (*r* = 0.58, *β* = 0.42, *p* < 0.001); SF‐36 (*r* = −0.58, *β* = −0.47, *p* < 0.001)	Medium/ Large	Medium
General distress						
Márki et al., 2017	Depression [hospital anxiety and depression scale] Anxiety [hospital anxiety and depression scale] Perceived stress [perceived stress scale]	HRQoL [36‐item short form survey]	Spearman *rho* correlation	HRQoL total (*rho* = −54, *p* < 0.001); physical HRQoL (*rho* = −0.33, *p* < 0.001); mental HRQoL (*rho* = −0.62, *p* < 0.001). HRQoL total (*rho* = −60, *p* < 0.001); physical HRQoL (*rho* = −0.42, *p* < 0.001); mental HRQoL (*rho* = −0.70, *p* < 0.001). HRQoL total (*rho* = −55, *p* < 0.001); physical HRQoL (*rho* = −0.34, *p* < 0.001); mental HRQoL (*rho* = −0.64, *p* < 0.001).	Large/ Medium	Medium
McPeak et al., 2018	Depression [patient health quality‐9] Anxiety [generalized anxiety disorder‐7]	HRQoL [Endometriosis health profile]	Spearman *rho* correlation	*rho* = 0.48, *p* < 0.001 *rho* = 0.29, *p* < 0.001	Large	Medium
Melis et al., 2015	Depression [Beck depression inventory] Anxiety [Beck anxiety inventory]	HRQoL [36‐item short‐form survey]	Spearman *rho* correlation	HRQoL total (*rho* = −65, *p* < 0.001); physical HRQoL (*rho* = −0.47, *p* < 0.001); mental HRQoL (*rho* = −0.64, *p* < 0.001). HRQoL total (*rho* = −60, *p* < 0.001); physical HRQoL (*rho* = −0.40, *p* < 0.001); mental HRQoL (*rho* = −0.60, *p* < 0.001).	Large/ Medium	Medium
Mundo‐López et al., 2020	Depression [hospital anxiety and depression scale] Anxiety	HRQoL [Endometriosis health profile]	Multivariate linear regression	*β* = 1.25, *p* < 0.001 *β* = 1.33, *p* < 0.001	‐	Medium
Roomaney et al., 2020	Depression [Beck depression inventory]	HRQoL [short form health survey; Endometriosis health profile]	Pearson's *r* correlation	Physical functioning (*r* = −40, *p* < 0.001); feelings about infertility (*r* = 0.20, *p* < 0.05); feelings about sexual relationships (*r* = 0.30, *p* < 0.001)	Medium/ Small	Medium
Sullivan‐Myers et al., 2021	Depression [Depression, Anxiety and Stress Scales] Anxiety Stress	HRQoL [Endometriosis health profile] [36‐item short form survey]	Pearson's *r* correlation	Pain (*r* = 0.35, *p* < 0.01); control/ powerlessness (*r* = 0.40, *p* < 0.01); social (*r* = 0.48, *p* < 0.01), self‐image (*r* = 0.38, *p* < 0.01). Physical function (*r* = −0.31, *p* < 0.01); role physical (*r* = −0.30; *p* < 0.01); vitality (*r* = −0.47, *p* < 0.01); social functioning (*r* = −0.49, *p* < 0.01). Pain (*r* = 0.38, *p* < 0.01); control/ powerlessness (*r* = 0.34, *p* < 0.01); social (*r* = 0.44, *p* < 0.01), self‐image (*r* = 0.36, *p* < 0.01). Physical function (*r* = −0.26, *p* < 0.01); role physical (*r* = −0.28; *p* < 0.01); vitality (*r* = −0.26, *p* < 0.01); social functioning (*r* = −0.38, *p* < 0.01). Pain (*r* = 0.32, *p* < 0.01); control/ powerlessness (*r* = 0.34, *p* < 0.01); social (*r* = 0.44, *p* < 0.01), self‐image (*r* = 0.37, *p* < 0.01). Physical function (*r* = −0.20, *p* < 0.01); role physical (*r* = −0.26; *p* < 0.01); vitality (*r* = −0.41, *p* < 0.01); social functioning (*r* = −0.42, *p* < 0.01).	Medium/ small	Medium
Petrelluzzi et al., 2012	Perceived stress [perceived stress questionnaire]	HRQoL [36‐item short form survey]	Pearson's *r* correlation	Vitality (*r* = −0.52, *CI* = ‐0.16, −0.76); other HRQoL subscales (*p*s >0.05)	Large	Medium
Petrelluzzi et al., 2008	Perceived stress [perceived stress questionnaire]	HRQoL [36‐item short‐form survey]	Spearman *rho* correlation	Mental HRQoL (*rho* = −0.52); other HRQoL subscales (*p*s >0.05)	Large	Medium
Emotional regulation						
Márki et al., 2017	Emotion regulation [difficulties in emotion regulation scale]	HRQoL [36‐item short form survey]	Spearman *rho* correlation	HRQoL total score (*rho* = −38, *p* < 0.001); physical HRQoL (*rho* = −0.17, *p* < 0.05); mental HRQoL (*rho* = −0.52, *p* < 0.001).	Medium	Medium
Melis et al., 2014	Alexithymia [Toronto alexithymia scale]	HRQoL [36‐item short‐form survey]	Pearson's *r* correlation	HRQoL total (*r* = −0.49, *p* < 0.001); physical function (*r* = −0.39, *p* < 0.001); general health (*r* = −0.35, *p* < 0.001); vitality (*r* = −41, *p* < 0.001); social functioning (*r* = −28, *p* < 0.001); role emotional (*r* = −39, *p* < 0.001); mental health (*r* = −0.49, *p* < 0.001); physical health (*r* = −39, *p* < 0.001).	Medium	Medium
Behavioural factors						
O'Hara et al., 2021	Number of self‐care activities [multiple choice question]	HRQoL [36‐item short form survey]	Linear regression	Physical HRQoL (*B* = ‐0.41, *p* < 0.05); mental HRQoL (*p* > 0.05)	‐	High
Social factors						
De Graaff et al., 2013	Partner present	HRQoL [36‐item short form survey]	Multivariate linear regression	Mental HRQoL (*β* = 0.14; *p* < 0.001); physical HRQoL (*p* > 0.05)	‐	High
Martins et al., 2021	Marital satisfaction [Couples Satisfaction Index]	HRQoL [Endometriosis health profile]	Pearson's *r* correlation Hierarchical multiple regression	HRQoL (*r* = 0.193; *p* < 0.05) HRQoL (*β* = −0.003, *p* > 0.05)	Small	High
O'Hara et al., 2021	Being in a stable relationship [multiple choice question]	HRQoL [36‐item short form survey]	Linear regression	Mental HRQoL (*B* = 3.51, *p* < 0.001); physical HRQoL (*p* > 0.05)	‐	High

### 4.1 General distress

#### 4.1.1 Depression

Six studies assessed the association between depression and pain in Endometriosis, only three of which found a significant association. Two studies reported that depression was associated with greater pain using regression (*β* = 0.19, *p* < 0.001) (Facchin et al., 2017) and correlation (*r* = 0.25, *p* < 0.01) (Laganà et al., 2015). Facchin et al. (2015) found that depression was associated with greater non‐menstrual pelvic pain (*β* = 0.47, *p* < 0.001), but not with dysmenorrhea, dyspareunia or dyschezia (*p*s >0.05). In the remaining three studies, depression was not associated with the presence of pelvic pain (Facchin et al., 2019), pain severity (Eriksen et al., 2008) or pain during intercourse (Mińko et al., 2021).

Six studies assessed the association between depression and HRQoL, all of which reported significant medium to large effect size associations. Melis et al. (2015) found that depression was linked with worse HRQoL total score (*rho* = −65, *p* < 0.001) and with impaired physical (*rho* = −0.47, *p* < 0.001) and mental HRQoL (*rho* = −0.64, *p* < 0.001). These findings were supported by Márki et al. (2017) who reported significant negative associations with HRQoL total score (*rho* = −54, *p* < 0.001), physical (*rho* = −0.33, *p* < 0.001) and mental HRQoL subscales (*rho* = −0.62, *p* < 0.001). Two other studies found that depression was associated with worse HRQoL, using correlation (*rho* = 0.48, *p* < 0.001) (McPeak et al., 2018) and linear regression which controlled for pain intensity (*β* = 1.21, *p* < 0.001; Mundo‐López et al., 2020). Roomaney et al. (2020) included various aspects of HRQoL assessed by the EHP‐30 (Jones et al., 2001) and found that depression was associated with worse physical functioning (*r* = −40, *p* < 0.001) and more negative feelings about infertility (*r* = 0.20, *p* < 0.05) and sexual relationships (*r* = 0.30, *p* < 0.001). Similarly, Sullivan‐Myers et al. (2021) found that depression was linked with multiple domains of HRQoL assessed by EHP‐30 (pain, *r* = 0.35; control/powerlessness, *r* = 0.40; social, *r* = 0.48; self‐image, *r* = 0.38; *ps* <0.01) and SF‐36 (physical function, *r* = −0.31; role physical, *r* = −0.30; vitality, *r* = −0.47; social functioning, *r* = −0.49; *ps* <0.01).

#### 4.1.2 Anxiety

Seven studies explored anxiety in relation to pain. Anxiety was linked with greater pelvic pain intensity (*β* = 0.285, *p* < 0.01) (Facchin et al., 2015), severity (*β* = 0.21, *p* < 0.001) (Facchin et al., 2017), but not with dysmenorrhea, dyspareunia or dyschezia (Facchin et al., 2015). Laganà et al. (2015) also found that anxiety was positively associated with pelvic pain (*r* = 0.26, *p* < 0.01) and dysmenorrhea (*r* = 0.16, *p* < 0.05). Sepulcri and Amaral (2009) reported that pain intensity was correlated with state anxiety (*p* < 0.01) and general anxiety (*p* < 0.001), whilst the association between pain and trait anxiety was not significant (*p* = 0.048). Nevertheless, in this study, the correlation coefficients were not mentioned. Facchin et al. (2019) found that the presence of pelvic pain was also linked with increased anxiety (*β* = 0.22, *r* ^2^ = 0.06, *p* < 0.01). Lastly, anxiety was also linked with greater pain during sexual intercourse (Mińko et al., 2021). Eriksen et al. (2008) did not find a significant correlation between anxiety and pain severity (*p* > 0.05).

Five studies explored the relationship between anxiety and HRQoL in Endometriosis, all of which found significant medium to large effect size associations. Two studies found that anxiety was linked with impaired HRQoL, using regression which controlled for pain (*β* = 1.29, *p* < 0.001) (Mundo‐López et al., 2020) and correlation (*rho* = 0.29, *p* < 0.001) (McPeak et al., 2018). In line with this, Melis et al. (2015) found significant correlations between anxiety and the total HRQoL score (*rho* = −0.60, *p* < 0.001), physical (*rho* = −0.40, *p* < 0.001) and mental HRQoL (*rho* = −0.60, *p* < 0.001). Márki et al. (2017) found that anxiety impairs general HRQoL (*rho* = −0.60, *p* < 0.001), physical HRQoL (*rho* = −0.42, *p* < 0.001) and mental HRQoL (*rho* = −0.70, *p* < 0.001). Finally, Sullivan‐Myers et al. (2021) reported that anxiety was impaired the following domains of HRQoL: pain (*r* = 0.38, *p* < 0.01), control/ powerlessness (*r* = 0.34, *p* < 0.01), social (*r* = 0.44, *p* < 0.01), self‐image (*r* = 0.36, *p* < 0.01), physical function (*r* = −0.26, *p* < 0.01), role physical (*r* = −0.28; *p* < 0.01), vitality (*r* = −0.26, *p* < 0.01) and social functioning (*r* = −0.38, *p* < 0.01).

#### 4.1.3 Stress

Petrelluzzi et al., (2008, 2012), did not find a significant association between perceived stress and pain (*p* > 0.05).

Four papers studied the relationship between perceived stress and HRQoL, all of which found significant associations Two studies indicated that perceived stress was associated with worse mental HRQoL (*rho* = −0.52, *p* < 0.05) and vitality (*r* = −0.52, *CI* = −0.16, −0.76) (Petrelluzzi et al., 2008, 2012). Perceived stress was linked with worse HRQoL total score (*rho* = −0.55, *p* < 0.001) and with impaired physical (*rho* = −0.34, *p* < 0.001) and mental HRQoL (*rho* = −0.64, *p* < 0.001) (Márki et al., 2017). In line with, Sullivan‐Myers et al. (2021) also reported that stress was linked with impaired HRQoL and was significantly correlated with multiple HRQoL domains: pain (*r* = 0.32, *p* < 0.01), control/powerlessness (*r* = 0.34, *p* < 0.01), social (*r* = 0.44, *p* < 0.01), self‐image (*r* = 0.37, *p* < 0.01), physical function (*r* = −0.20, *p* < 0.01), role physical (*r* = −0.26; *p* < 0.01), vitality (*r* = −0.41, *p* < 0.01) and social functioning (*r* = −0.42, *p* < 0.01).

#### 4.1.4 Generalized worry

One study explored the association between pain and generalized worry, in which a positive correlation was found (*r* = 0.32, *p* < 0.001) (Zarbo et al., 2019).

#### 4.1.5 Somatisation

One included study reported that somatisation, defined as “the tendency to experience psychological distress in the form of somatic symptoms” (Lipowski, 1987), was positively associated with pelvic pain (*r* = 0.31, *p* < 0.01) (Laganà et al., 2015).

### 4.2 Cognitive factors

#### 4.2.1 Catastrophising

Three studies explored the association between pain and catastrophising in Endometriosis. Zarbo et al. (2019) found that catastrophising was linked with greater pain at baseline (*r* = 0.268, *p* < 0.001), whilst Martin et al. (2011) reported that baseline catastrophising predicted pain severity at 12 months (*β* = 0.18, *p* < 0.05, *r* ^2^ = 0.03). In both studies, the effect size of the association was small. In another study (Carey et al., 2014), catastrophising was not associated with the total pain score (*p* = 0.055) but with the affective pain score (*β* = 0.66, *p* < 0.01).

One study reported that catastrophising was associated with worse HRQoL using correlation (*rho* = 0.56, *p* < 0.001) (McPeak et al., 2018) and regression (*β* = 1.47, *p* < 0.001) (Mundo‐López et al., 2020).

#### 4.2.2 Rumination

Facchin et al. (2017) found that rumination was associated with greater pelvic pain severity (*β* = 0.25, *p* < 0.001).

#### 4.2.3 Self‐blame

Zarbo et al. (2019) reported a small positive correlation between self‐blame and pain severity (*r* = 0.15, *p* < 0.001).

#### 4.2.4 Self‐efficacy

One study found that self‐efficacy, defined as “someone's belief that they are able to carry out a behaviour” (Bandura, 1977), was associated with improved physical (*B* = 1.30, *p* < 0.001) and mental HRQoL (*B* = 1.55, *p* < 0.001), after controlling for demographic characteristics and pain (O'Hara et al., 2021).

#### 4.2.5 Beliefs about pain control

One study (Bylinka & Oniszczenko, 2016) found that beliefs about internal control of pain (i.e., the individual's belief that they can control pain) were linked with greater pain intensity (*r* = −0.31, *β* = −0.24, *p* < 0.01). Doctor control beliefs (i.e., beliefs that doctors can control pain) were correlated with more pain (*r* = 0.33, *p* < 0.01), however, the regression analysis was not significant (*p* > 0.05). Beliefs that pain is controlled by chance events (e.g., belief that being in pain depends on luck) were not associated with pain (*p* > 0.05).

van Aken et al. (2017) found that pain cognition was associated with impaired HRQoL, measured by the EHP‐30 (*r* = 0.58, *β* = 0.42, *p* < 0.001) and SF‐36 (*r* = 0.58, *β* = 0.42, *p* < 0.001). van Aken et al. (2017) defined pain cognition as “the psychological aspects of pain catastrophising, pain anxiety and pain vigilance.” This was measured using the average z scores of these three questionnaires.

#### 4.2.6 Illness acceptance

Andysz and Merecz‐Kot (2021) explored illness acceptance in relation to HRQoL and found that after controlling for baseline variables (e.g., infertility, pain catastrophising), acceptance was linked with better pain‐related HRQoL (*β* = −0.27, *p* < 0.001). More specifically they used the pain subscale of EHP‐30, which assesses the effect of pain on the patient's life.

#### 4.2.7 Body attitude

One included study explored the association between attitudes towards body and HRQoL and found negative medium to large effect size associations (Melis et al., 2015). More specifically, negative appreciation of the body, lack of familiarity with own body and general body dissatisfaction were associated with worse total HRQoL (*rho* = −37, *rho* = −59, *rho* = −43, *ps* <0.001, respectively) and with impaired physical HRQoL (*rho* = −0.38, *rho* = −0.58, *rho* = −0.39, *ps* <0.001, respectively) and mental HRQoL (*rho* = −0.31, *rho* = −0.49, *rho* = −0.35, *ps* <0.001, respectively).

#### 4.2.8 Self‐esteem

One study indicated that the presence of pelvic pain was associated with lower self‐esteem (*β* = −0.23, *r* ^2^ = 0.06, *p* < 0.01) (Facchin et al., 2019).

### 4.3 Emotion regulation factors

#### 4.3.1 Emotion regulation

Márki et al. (2017) found that difficulties in emotion regulation had a medium size negative correlation with HRQoL total score (*rho* = −38, *p* < 0.001) and with physical (*rho* = −0.17, *p* < 0.05) and mental HRQoL (*rho* = −0.52, *p* < 0.001).

#### 4.3.2 Alexithymia

Alexithymia refers to the difficulty of describing emotions and feelings (Lesser, 1981). This was linked with greater pelvic pain intensity (*r* = 0.35, *p* < 0.05), whilst dyspareunia was negatively correlated with an alexithymia subscale, difficulty identifying feelings (*r* = −0.35, *p* < 0.05), but not with alexithymia total score (*p* = 0.56) (Cavaggioni et al., 2014).

Alexithymia had a medium size negative correlation with HRQoL total score (*r* = −0.49, *p* < 0.001) and with the subscales: physical function (*r* = −0.39, *p* < 0.001); general health (*r* = −0.35, *p* < 0.001); vitality (*r* = −41, *p* < 0.001); social functioning (*r* = −28, *p* < 0.001); role emotional (*r* = −39, *p* < 0.001); mental health (*r* = −0.49, *p* < 0.001); and physical health (*r* = −39, *p* < 0.001) (Melis et al., 2014).

### 4.4 Behavioural factors

Interestingly, a greater number of self‐care activities was associated with worse physical HRQoL (*B* = ‐0.41, *p* < 0.05) but not with mental HRQoL (*p* > 0.05) (O'Hara et al., 2021). The authors assessed activities that people used to manage symptoms of Endometriosis, such as changing diet, exercising or taking supplements.

### 4.5 Personality

#### 4.5.1 Temperament and character traits

Two studies tested the association between temperament/personality traits and pain intensity/severity in Endometriosis. Facchin et al. (2016) found that harm avoidance, defined as “the tendency to be fearful, worried and sensitive to criticism” (Naylor et al., 2017), was linked with greater pelvic pain (*B* = 0.08; *p* < 0.01). Conversely, self‐directedness, defined as “someone's belief that they can control their life and solve their problems” (Farmer & Goldberg, 2008), was associated with less pain (*B* = ‐0.05; *p* < 0.05). Bylinka and Oniszczenko (2016) reported that endurance, which refers to “the ability to go through challenging or demanding situations” (Strelau & Zawadzki, 1993) was associated with greater pain intensity (*r* = −0.51; *β* = 0.56, p < 0.001).

### 4.6 Social factors

None of the included studies assessed social factors in relation to pain in Endometriosis.

Three studies explored the association between social/dyadic factors and HRQoL. O'Hara et al. (2021) found that being in a stable relationship was associated with improved mental HRQoL (*B* = 3.51, *p* < 0.001) but not with physical HRQoL (*p* > 0.05). Similarly, De Graaff et al. (2013) reported that after controlling for pain and number of comorbidities, having a partner present was linked with better mental (*β* = 0.14; *p* < 0.001), but not physical (*p* > 0.05) HRQoL. Martins et al. (2021) found that greater marital satisfaction was correlated with improved HRQoL (*r* = 0.193; *p* < 0.05), however this association was not significant after controlling for covariates (e.g., psychological comorbidity) in regression (*β* = −0.003, *p* > 0.05).

## 5 DISCUSSION

This is the first systematic review, to our knowledge, to explore the association between psychosocial factors with pain and HRQoL in Endometriosis. Pain and HRQoL were the two outcomes studied. The psychosocial factors most frequently explored were depression, anxiety and catastrophising. Many psychosocial factors were only researched once, and there was scarce research into social factors.

### 5.1 Psychological factors associated with pain

The positive association between anxiety and pain found in this review is consistent with systematic reviews in general pelvic pain (Lagana et al., 2017; Riegel et al., 2014; Vitale et al., 2020). However, the included studies that examined anxiety and pain were cross‐sectional, so the direction of association is unclear. Conversely, the associations between depression and pain were mixed, since three studies reported significant positive associations, and three studies did not. Similar to anxiety, all these studies were cross‐sectional. These findings are surprising since previous systematic reviews have shown significant associations between depression and pelvic pain in Endometriosis and Vulvodynia (Chisari, Monajemi, et al., 2021; Gambadauro et al., 2019; Pope et al., 2015).

A possible explanation for these mixed results is that a confounder, such as cognitive appraisal or coping behaviours, could disrupt the association between affective outcomes and pain in Endometriosis, and none of these studies controlled for confounders. Research in persistent pain indicates that patients' appraisal of the impact and controllability of pain mediates the association between depression and pain (Gatchel et al., 2007; Turk et al., 1995). Furthermore, other unmeasured psychosocial variables may mask the association between depression and pain. Eriksen et al. (2008) reported that emotion suppression, which is prevalent in Endometriosis (Zarbo et al., 2018), could result in the underreporting of depressive symptoms and mask the association between depression and pain. Stigma is another factor that could mediate the association between pain invalidation and depression (Boring et al., 2021). Further research, which controls for confounders and has longitudinal designs, is needed to elucidate the relationship between depression and pain in this population.

In addition to anxiety and depression, catastrophising was linked with greater pain in three studies, one of which was longitudinal (Martin et al., 2011). The positive association between catastrophising and pelvic pain is consistent with previous systematic reviews (Chisari, Monajemi, et al., 2021; Huang et al., 2020). The role of catastrophising in persistent pain has been highlighted in the fear‐avoidance model (Vlaeven et al., 2016). According to this, catastrophic interpretations of pain symptoms could increase physiological arousal, avoidance behaviour and fear, which could, in turn, increase pain and disability (Gatchel et al., 2007; Vlaeven et al., 2016). Fear‐avoidance has been associated with pain in women with chronic pelvic pain and sexual pain (Gatchel et al., 2007; Thomtén et al., 2014). Previous studies have also found that catastrophising mediates the association between depression and pain (Kim et al., 2021; Wood et al., 2013). The mediating role of catastrophising may further explain the mixed findings earlier discussed regarding the association between depression and pain in Endometriosis.

The review also found that other psychological factors were associated with pain; however, these were explored only once and therefore, their implications may be limited. For instance, rumination, alexithymia and beliefs about internal control of pain were all associated with greater pain in Endometriosis. Previous research on the role of perceived pain control (Gatchel et al., 2007) and alexithymia (Di Tella & Castelli, 2016) in persistent pain showed mixed results. As for rumination, previous research has found a positive correlation between rumination and pain severity, although this was not significant in regression analysis (Craner et al., 2016). Lastly, endurance and self‐directedness were associated with decreased pain, whereas harm avoidance was linked with greater pain. A critical review suggests that people living with persistent pain present higher harm avoidance and lower self‐directedness compared to controls (Naylor et al., 2017), and these coping strategies may in turn increase or decrease pain levels.

### 5.2 Psychological factors and health‐related quality of life

Overall, the studies of the review found that depression and anxiety were associated with worse HRQoL. These findings are in line with previous systematic reviews, according to which, depression and anxiety negatively impact HRQoL in other long‐term health conditions (Ali et al., 2010; Blakemore et al., 2014). Catastrophising was also linked with worse HRQoL in this review; this was found to partially mediate the association between pelvic pain and HRQoL in Endometriosis (Mundo‐López et al., 2020). The mediating effect of catastrophising on the association between pain and HRQoL has also been found in Fibromyalgia (Galvez‐Sánchez et al., 2020).

A range of psychological factors in relation to HRQoL was only explored once in Endometriosis. These include negative body attitude, alexithymia and difficulties in emotion regulation, which were associated with impaired HRQoL. Previous research in persistent pain indicates that alexithymia is associated with reduced functioning (Di Tella & Castelli, 2016), and emotion regulation with impaired quality of life (Agar‐Wilson & Jackson, 2012). Similarly, body image has been linked with worse quality of life in Fibromyalgia (Akkaya et al., 2012). One study found that illness acceptance was associated with improved HRQoL, even after controlling for covariates. Acceptance of pain has also been linked with greater quality of life in people with chronic back pain (Mason et al., 2008). Lastly, this review found that self‐efficacy was linked with improved HRQoL, which is confirmed by previous research in persistent pain (Gatchel et al., 2007).

### 5.3 Social factors associated with pain and health‐related quality of life

Overall, social factors were narrowly researched. None of the included studies assessed social factors in relation to pain, whilst only three studies assessed the association between social/dyadic factors and HRQoL. More specifically, the presence of a partner was associated with better mental, but not physical HRQoL (De Graaff et al., 2013; O'Hara et al., 2021), whilst marital satisfaction was not significantly associated with HRQoL after controlling for covariates (Martins et al., 2021). Findings indicate that relationship status and satisfaction may influence only the mental component of HRQoL. Another included study found that being in a stable relationship was associated with reduced rumination in Endometriosis, whilst rumination was linked with greater pain (Facchin et al., 2017). The lack of existing research on the association of social factors with pain and HRQoL was surprising, considering the clear interpersonal and social impact of genito‐pelvic pain (Rosen & Bergeron, 2019). Indeed, social and interpersonal factors influence a range of physiological and psychological outcomes in persistent pain (Gatchel et al., 2007) and Vulvodynia (Bergeron & Rosen, 2020). The significant psychosocial impact of Endometriosis has also been highlighted in qualitative research, which showed that Endometriosis disrupts the personal identity of women (Cole et al., 2021). Furthermore, a significant social context for women with endometriosis is the experience within the healthcare system. This affects the psychological experience of women, who may feel disbelieved, uncertain and unsafe with no guarantee of support for debilitating symptoms (Sims et al., 2021; Tewhaiti‐Smith et al., 2022). It also can directly affect physiological outcomes due to factors like delay in diagnosis and lack of access to treatment (Tewhaiti‐Smith et al., 2022). Thus, further research is needed to investigate the association between social factors and pain and interference in this population.

## 6 IMPLICATIONS FOR CLINICAL PRACTICE AND FUTURE RESEARCH

**FIGURE 2:**
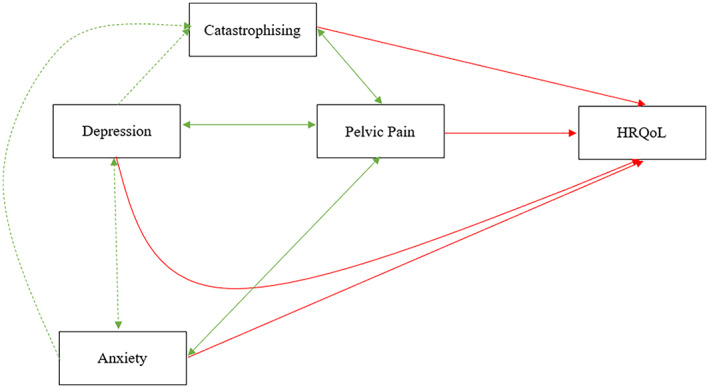
Summary figure of psychosocial factors in relation to pain and HRQoL in endometriosis. Green arrows represent positive associations; red arrows negative associations; double arrows bidirectional associations; solid arrows associations found in women with endometriosis; dotted arrows associations are hypothetical associations. Depression is associated with greater pain and worse HRQoL, whilst pain also impairs HRQoL. There is a bidirectional association between pain and depression, and pain and catastrophising. Note 1: HRQoL: Health‐related quality of life Note 2. Green arrows represent positive associations; red arrows represent negative associations; double arrows represent bidirectional associations; solid arrows reflect associations found in the included studies of women with Endometriosis; dotted arrows display hypothetical associations. Note 3: Depression is associated with greater pain and worse HRQoL, whilst pain also impairs HRQoL. There is a bidirectional association between pain and depression, and pain and catastrophising.

To better represent the findings of this review and orient future research, a summary figure is provided (Figure 2). This highlights the role of depression, anxiety and catastrophising in pain and HRQoL. Given the relevance of these factors, efforts to improve emotional functioning may lead to improvements in pain and quality of life in this population. Future research and clinicians could explore psychosocial approaches that may improve emotional functioning, reduce pain impact, and enhance women's quality of life, such as Cognitive Behavioural Therapy and Acceptance and Commitment Therapy. These have been successfully applied to persistent pain (Trindade et al., 2021; Williams et al., 2020), suggesting their potential in Endometriosis.

This review also highlights the lack of existing research into social factors. The lack of social factors calls for more research in the field. Previous research has indicated that experiences of dismissal have been reported by women with Endometriosis (Cox et al., 2003). Similarly, women with Endometriosis report that clinicians are not willing to understand their individual needs (Lamvu et al., 2020). This is consistent with past research that also highlights that pain symptoms expressed by women are more often underestimated (Samulowitz et al., 2018; Zhang et al., 2021). This suggests social and systemic factors need further exploration and consideration, as potentially relevant constructs such as perceived injustice (Sullivan et al., 2008) have yet to be applied to Endometriosis.

Lastly, research on protective factors for pain and HRQOL (e.g., cognitive flexibility, acceptance) needs further research. This would also help clinicians focus on what is important for women with Endometriosis, potentially reducing distress and impact.

## 7 LIMITATIONS

This systematic review presents limitations. Firstly, all studies except one were cross‐sectional, which means that causal relationships cannot be inferred. Research on social and dyadic factors, especially in relation to pain in Endometriosis was very limited. Given that most studies did not include confounders in the regression analysis, any associations found could be partially or completed attributed to other variables. Studies did not include women under the age of 17 and most of them were conducted in developed Western countries. The authors were unable to access unpublished studies or translate non‐English papers, which may have led to increased publication bias.

## 8 CONCLUSIONS

The systematic review explored psychosocial factors in relation to pain and HRQoL in Endometriosis. The review indicates that catastrophising and anxiety are associated with greater pain, whereas anxiety and depression are related to worse HRQoL. Research on other psychosocial factors, particularly social variables needs further exploration. Nevertheless, the review findings indicate that a psychosocial conceptualisation of Endometriosis is supported. Targeting depression, anxiety, and catastrophising in future treatments of women with Endometriosis may lead to improved outcomes and is therefore warranted.

## AUTHOR CONTRIBUTIONS

Michail Kalfas: draft protocol, develop and perform search strategy, obtain full‐text reports, carry out study screening, interpret findings and draft final review. Claudia Chisari: draft protocol, develop search strategy, carry out study screening, interpret findings and draft final review. Sula Windgassen: draft protocol, develop search strategy, carry out study screening, interpret findings and draft final review. All authors discussed the results and commented on the manuscript.

## FUNDING INFORMATION

C.C. was funded by the National Institute for Health Care (NIHC) Biomedical Research Centre at South London and Maudsley NHS Foundation Trust and King's College London in the form of a PhD Studentship. The views expressed are those of the authors and not necessarily those of the National Health Service, the NIHR or the Department of Health and Social Care.

## CONFLICT OF INTEREST

None to declare.

## Supporting information


Appendix S1
