# Supplementary material for: Psychosocial factors associated with pain and health‐related quality of life in Endometriosis: A systematic review
Source: Eur J Pain. 2022 Jul 22;26(9):1827–48. doi: 10.1002/ejp.2006 (PMC9543695; doi:10.1002/ejp.2006)
Supplement: Supplementary file 1 — Appendix S1 [file EJP-26-1827-s001.docx]

**Appendices**

**Appendix S1**

**Table S1. Search terms**

| exp endometriosis/ or endometriosis.mp | exp pain/ or pain.mp | exp psychological factors/ or psychological factors.mp |
| --- | --- | --- |
|  | (dysmenorrhea or dyspareunia or dysuria or dyschezia).mp | psycholog*.mp |
|  | exp disease severity/ or disease severity.mp | (depress* or major depressive disorder).mp |
|  | exp symptom severity/ or symptom severity.mp | anxiety.mp |
|  |  | stress.mp |
|  |  | distress.mp |
|  |  | cognit*.mp |
|  | interference.mp | beliefs.mp |
|  | exp health-related quality of life/ or health-related quality of life. mp | Percept* .mp |
|  | 11. Or/2-7 | emotion*.mp |
|  |  | affective.mp |
|  |  | mood.mp |
|  |  | coping.mp |
|  |  | accept*.mp |
|  |  | (behaviour* or behavior*).mp |
|  |  | personality.mp |
|  |  | castrophi?ing.mp |
|  |  | avoid*.mp |
|  |  | exp social factors/ or social factors.mp |
|  |  | social function.mp |
|  |  | social support mp |
|  |  | dyadic factors.mp |
|  |  | interpersonal factors.mp |
|  |  | 35. Or/7-31 |

**Appendix S2**

***Table S2. Adapted CASP checklist questions***

| 1) Did the study address the association of psychosocial factors with painor health-related quality of life in endometriosis? |
| --- |
| 2) Did the authors use an appropriate method to answer their research question(s)? |
| 3) Were women with endometriosis recruited in an acceptable way? |
| 4) Was the outcome accurately measured to minimise bias? |
| 5) Have the authors taken into account any confounding factor(s) in the analysis? |
| 6) Was there a follow-up of participants? |
| 7) Was the follow-up long enough? |
| 8) Do the authors present important theoretical or practical implications of their findings? |

*Response scale: Yes; Can’t tell; No.*

**Appendix S3**

*Table S3. Risk of bias assessment using the adapted CASP checklist for each study.*

| Reference | Question 1 | Question 2 | Question 3 | Question 4 | Question 5 | Question 6 | Question 7 | Question 8 | Quality Score |
| --- | --- | --- | --- | --- | --- | --- | --- | --- | --- |
| Andysz & Merecz-Kot, 2021 | Yes | Yes | Yes | Yes | Yes | No | - | Yes | High |
| Bylinka & Oniszczenko, 2016 | Yes | Yes | Yes | Yes | No | No | - | Yes | Medium |
| Carey et al., 2014 | Yes | Yes | Yes | Yes | No | No | - | Yes | Medium |
| Cavaggioni et al., 2014 | Yes | Yes | Yes | Yes | No | No | - | Yes | Medium |
| De Graaff et al., 2013 | Yes | Yes | Yes | Yes | Yes | No | - | Yes | High |
| Eriksen et al., 2008 | Yes | Yes | Yes | Yes | No | No | - | Yes | Medium |
| Facchin et al., 2015 | Yes | Yes | Yes | Yes | No | No | - | Yes | Medium |
| Facchin et al., 2016 | Yes | Yes | Yes | Yes | No | No | - | Yes | Medium |
| Facchin et al., 2017 | Yes | Yes | Yes | Yes | No | No | - | Yes | Medium |
| Facchin et al., 2019 | Yes | Yes | Yes | Yes | No | No | - | Yes | Medium |
| Lagana et al., 2015 | Yes | Yes | Yes | Yes | No | No | - | No | Medium |
| Marki et al., 2017 | Yes | Yes | Yes | Yes | No | No | - | Yes | Medium |
| Martin et al., 2011 | Yes | Yes | Yes | Yes | Yes | Yes | Yes | Yes | High |
| Martins et al., 2021 | Yes | Yes | Yes | Yes | Yes | No | - | Yes | High |
| McPeak et al., 2017 | Yes | Yes | - | Yes | No | No | - | Yes | Medium |
| Melis et al., 2014 | Yes | Yes | Yes | Yes | No | No | - | Yes | Medium |
| Melis et al., 2015 | Yes | Yes | Yes | Yes | No | No | - | Yes | Medium |
| Minko et al., 2021 | Yes | Yes | Yes | Yes | No | No | - | Yes | Medium |
| Mundo-Lopez et al., 2020 | Yes | Yes | Yes | Yes | No | No | - | Yes | Medium |
| O’Hara et al., 2021 | Yes | Yes | Yes | Yes | Yes | No | - | Yes | High |
| Petrelluzi et al., 2008 | Yes | Yes | Yes | Yes | No | No | - | No | Medium |
| Petrelluzi et al., 2012 | Yes | Yes | - | Yes | No | No | - | Yes | Medium |
| Roomaney et al., 2019 | Yes | Yes | Yes | Yes | No | No | - | Yes | Medium |
| Sepulcri & Amaral, 2008 | Yes | Yes | Yes | Yes | No | No | - | No | Medium |
| Sullivan-Myers et al., 2021 | Yes | Yes | Yes | Yes | No | No | - | Yes | Medium |
| Van Aken et al., 2017 | No | Yes | Yes | Yes | No | No | - | Yes | Medium |
| Zarbo et al., 2019 | Yes | Yes | Yes | Yes | No | No | - | Yes | Medium |

*a. Cut-off scores: 0-2= low quality; 3-5=medium quality; 6-8=high quality*
